# A Nomogram to Predict Regrowth After Ultrasound-Guided Radiofrequency Ablation for Benign Thyroid Nodules

**DOI:** 10.3389/fendo.2021.774228

**Published:** 2022-02-17

**Authors:** Lin Yan, Mingbo Zhang, Xinyang Li, YingYing Li, Yukun Luo

**Affiliations:** Department of Ultrasound, First Medical Center, Chinese PLA General Hospital, Beijing, China

**Keywords:** ablation techniques, radiofrequency ablation, ultrasonography, nomograms, thyroid nodule

## Abstract

**Objective:**

To develop and validate a nomogram to predict regrowth for patients with benign thyroid nodules undergoing radiofrequency ablation (RFA).

**Methods:**

A total of 200 patients with 220 benign thyroid nodules who underwent RFA were included in this respective study. After RFA, patients were followed up at 1, 3, 6, and 12 months, and every 12 months thereafter. Regrowth was defined as an increase in nodule volume 50% over the previously recorded smallest volume. A nomogram was developed based on the variables identified by multivariate logistic regression and the model performance was evaluated by discrimination(concordance index) and calibration curves.

**Results:**

The incidence of regrowth was 13.64% (30/220) after a mean follow-up period of 27.43 ± 17.99 months. Multivariate logistic regression revealed initial volume (OR = 1.047, 95%CI 1.020–1.075), vascularity (OR = 2.037, 95%CI 1.218–3.404), and location close to critical structure (OR = 4.713, 95%CI 1.817–12.223) were independent factors associated with regrowth. The prognostic nomogram incorporating these three factors achieved good calibration and discriminatory abilities with a concordance index of 0.779 (95%CI 0.686–0.872).

**Conclusions:**

A prognostic nomogram was successfully developed to predict nodule regrowth after RFA, which might guide physician in stratifying patients and provide precise guidance for individualized treatment protocols.

## Introduction

Thyroid nodules are a common finding, present in up to 65% of the general population (1). Most nodules are benign and asymptomatic; however, a minority may require treatment because of the compressive symptomatic or cosmetic problems. Surgery is the standard treatment, which can remove a nodule completely at the cost of potential risk of complications, scar formation, and damage to normal thyroid parenchyma (2, 3). Thus, there has been a growing interest in developing the minimally invasive treatment that is much safer and allows gland retention for the treatment of benign thyroid nodules.

Ultrasound (US)-guided thermal ablation techniques, namely, radiofrequency ablation (RFA), microwave ablation (MWA), and laser ablation (LA) have been recommended as alternatives to surgery for benign thyroid nodules (3–7). These thermal ablation techniques have been successfully applied in treating patients with benign thyroid nodules (8–16). A recent meta-analysis included 24 studies on ablation and found that the volume reduction rates (VRR) at 6, 12, 24, and 36 months during the follow-up period were 60, 66, 62, and 53%, respectively (17). Moreover, the major complications rate was only 1.3% without life-threatening adverse events (18). Although satisfactory results were achieved, several studies found that approximately 5.6–38% of the treated nodule occurred regrowth after 2 to 3 years after ablation (19–23). Regrowth was defined as ≥50% volume increase compared to the previously recorded smallest volume (24). It usually occurred from the untreated peripheral area and additional ablation might be beneficial (19, 20, 22, 25, 26). Therefore, early evaluation of the risk of regrowth after ablation for benign thyroid nodules was essential for the treatment planning and follow-up strategy. Previous studies found that when dividing the total volume of ablated nodule after ablation into the ablated volume (Va) and the incompletely vital volume (Vv), an increased Vv could be an early sign of nodule regrowth (20, 27). Several other predictors were also reported to be associated with regrowth, namely, the 12-month VRR (23), energy applied per volume (EPV) (21), and residual vital ratio (RVR) (27). Because they could be only obtained during the follow-up after ablation, none of them could be used as an early predictor for nodule regrowth. Negro et al. (28) recent developed a machine learning algorithm to discriminate nodules with a VRR >50% at 12 months after RFA to identify the best candidates for effective treatment in one single session. However, accurate prediction of nodule regrowth after ablation is still lacking. A nomogram has been considered to be evidence-based, individualized and accurate in risk estimation, which has been applied to various malignancies (29–32). It has also been used to predict the clinical outcomes of patients with hepatocellular carcinoma who underwent thermal ablation (33–36). However, to our best knowledge, a prognostic nomogram for benign thyroid nodules following RFA has not yet been reported.

Therefore, the purpose of this study was to construct a nomogram to predict regrowth for patients with benign thyroid nodules undergoing RFA.

## Materials and Methods

Approval for this retrospective study was obtained from the Institutional Review Board of our institution (No. S2019-2111-01). Written information consent was obtained from all the patients prior to RFA procedure.

### Patients

The study inclusion criteria were as follows: (1) nodules confirmed as benign *via* two separated fine-needle aspiration or core-needle biopsy; (2) nodules without suspicious malignant features on US examination, namely, very hypoechoic, irregular margin, taller than wide, microcalcification, evidence of extrathyroidal extension, and suspicious cervical lymph node; (3) patients with solid(≤10% of fluid component) or predominantly solid nodules (11–50% of fluid component) (24); (4) patients who were complained about cosmetic, symptomatic problems or rapid growth; (5) serum thyroid hormone and thyrotropin levels within normal ranges; (6) patients who were refusal or ineligibility for surgical treatment; and (7) follow-up time was larger than 6 months. Exclusion criteria were: (1) malignancy findings or follicular neoplasm by biopsy; (2) nodules with benign result by biopsy had suspicious of malignancy on US examination; and (3) follow-up time was less than 6 months.

We retrospectively searched the database for patients who would be eligible for this study and selected the period from August 2014 to December 2018. After the exclusion of patients, 200 patients with 220 benign thyroid nodules with full medical records were included in this study.

### Pre-Ablation Assessment

US were performed using a Siemens Acuson Sequoia 512 Ultrasound System (Siemens, Mountain View, CA, USA) or a Philips iU22 Ultrasound System (Philips Healthcare, Bothell, WA) or a Mindray M9 Ultrasound System (Mindray, Shenzhen, China). Before RFA, patients underwent US, biopsy, clinical evaluation, and also the laboratory examination. The nodule volume was calculated by ellipsoid formula: V = πabc / 6 (V, nodule volume; a, the largest nodule diameter; b and c, the other two perpendicular diameters of the nodule). Nodule location was classified as normal location and close to critical structures (less than 2 mm), namely, trachea, cervical carotid artery, jugular vein, esophagus and recurrent laryngeal nerve. According to the component, the nodules were categorized as solid (≤10% of fluid component) and predominantly solid (11–50% of fluid component) (24). Nodule vascularity was classified using a 1–4 grade scale (4): grade 1, no vascularity; grade 2, peripheral vascularity; grade 3, intra-nodular vascularity<50%; and grade 4, intra-nodular vascularity ≥50%. The nodule-related symptom score was self-measured by the patient using a 10-cm visual analogue scale (grades 0–10) (4). The cosmetic score was evaluated by the physician as follows: 1, no palpable mass; 2, no cosmetic problem but palpable mass; 3, a cosmetic problem on swallowing only; and 4, a readily detected cosmetic problem) (4). The laboratory examination included a complete blood count, thyroid function tests, and also the blood coagulation tests.

### Ablation Procedure

One experienced US physician performed all RFA procedures using a bipolar RFA generator (CelonLabPOWER, Olympus Surgical Technologies Europe) and an 18-gauge bipolar RF electrodes with 0.9 cm active tip (CelonProSurge micro 100-T09, Olympus Surgical Technologies Europe).

Patients were in supine position with their necks extended. RFA was performed using the trans-isthmic approach and moving-shot technique after local anesthesia with 1% lidocaine. If the distance between the nodule and surrounding critical structures was <5 mm, hydrodissection technique was performed with injection of normal saline to prevent thermal injury. The output RFA power was 3–9 W. Contrast-enhanced ultrasound (CEUS) was conducted immediately after the RFA procedure to evaluate the ablation area. CEUS was performed after bolus injection of SonoVue (2.4 ml, Bracco), followed by a 5 ml of normal saline flush. If any enhancement existed in the treated nodule, a supplementary ablation was performed. The patients were observed for 1-2 h to check for possible adverse events or side effects.

### Post-Ablation Evaluation

After ablation, patients were followed up at 1, 3, 6, and 12 months and every 12 months thereafter and the volume, VRR, cosmetic and symptom scores were evaluated. VRR was calculated as follows: VRR = [(initial volume − final volume) × 100%] / initial volume (24). A >50% volume reduction at 12 months after ablation was defined as technical efficacy (24). Regrowth was defined as an increase in total volume 50% over the previously recorded smallest volume (24). According to the detection of regrowth, the nodules were divided into non-regrowth group and regrowth group. To exclude neoplastic transformation, CNB was performed to all the regrowth nodules, which was performed by a disposable 1.5- or 2.2-cm excursion, 20-gauge double-action spring-loaded needle (BARD Magumn, Bard Peripheral Vascular, Inc.) to the vital area of the nodule.

### Statistical Analysis

Continuous variables were reported as mean ± SD and categorical variables were expressed as numbers with percentages. Mann–Whitney U test were used to compare volume, VRR, symptom and cosmetic scores between the two groups. Univariate and multivariate logistic regression analysis of independent factors influencing regrowth were assessed, and the odds ratios (OR) with 95% confidence intervals (CI) were reported. A nomogram incorporated these independent factors was constructed to predict the probability of regrowth after RFA. The model performance was evaluated by discrimination and calibration (29). The discrimination of the nomogram was evaluated by the concordance index (C-index), which was equivalent to the area under the receiver operating characteristic curve. The value of the C-index varied between 0.5 and 1.0, with 1.0 indicating the perfect ability to correctly discriminate outcomes, and 0.5 indicating a random chance. Model validation was performed using bootstrap validation method with 1,000 resamples to quantify the overfitting of modeling strategy and predict future performance of the model (29). Calibration was evaluated using a calibration curve, which was a graphic representation of the relationship between the observed outcome frequencies and the predicted probabilities, with 1,000 bootstrap resamples of the study group. In a well-calibrated model, the predictions should fall on a 45-degree diagonal line. Statistical analyses were performed by using SPSS statistical software(Version 25.0) and R software version 3.6.2 (R Foundation for Statistical Computing). A two-sided *P* <0.05 was considered as statistically significant.

## Results

In this study, 200 patients (176 females, 24 males, mean age 46.02 ± 11.95 years) with 220 benign thyroid nodules were enrolled (**Table 1**). Among these 200 patients, 183 patients had 1 nodule, 14 patients had 2 nodules and 3 patients had 3 nodules. The initial volume was 10.30 ± 13.41 ml and the largest diameter was 3.00 ± 1.37 cm. During RFA, the mean power of was 6.83 ± 3.11 W. The mean energy was 2,639.32 ± 2,173.70 J and the mean EPV was 648.04 ± 678.55 J/ml.

**Table 1:** Clinical characteristics.

Characteristics	Data
No. of patients/nodules	200/220
Age (years)	46.02 ± 11.95
Sex (F/M)	176/24 (88.0/12.0)
Component	
Solid/ Predominately solid	155/65 (70.5/20.5)
Location	
Right lobe/Left lobe/Isthmus	114/104/2 (51.82/47.27/0.91)
Largest diameter (cm)	3.00 ± 1.37
Initial volume (ml)	10.30 ± 13.41
Vascularity	2.5 ± 0.9

Data are presented as mean ± SD or number of nodules (percentages).

The mean follow-up time was 27.43 ± 17.99 months. The mean VRR was 88.82 ± 11.98% and the technical efficacy was 96.82% (213/220). Symptom score significantly decreased from 2.72 ± 2.15 to 0.95 ± 1.18 (*P* <0.001). Cosmetic score significantly decreased from 2.45 ± 1.22 to 1.34 ± 0.58 (*P* <0.001).

**Figure 1:**
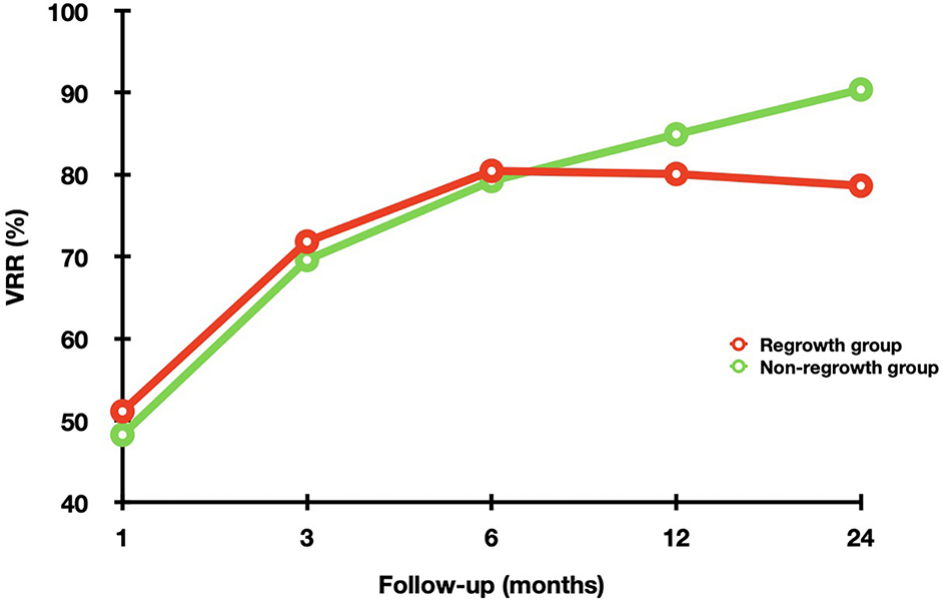
The changes of VRR in the non-regrowth and regrowth groups at each follow-up point after RFA. In the first 12 months, VRR in the two groups were nonsignificant (all *P* >0.05). However, at 24 months after RFA, VRR in the non-regrowth group were significantly larger than that in the regrowth group (*P* = 0.002).

**Figure 2:**
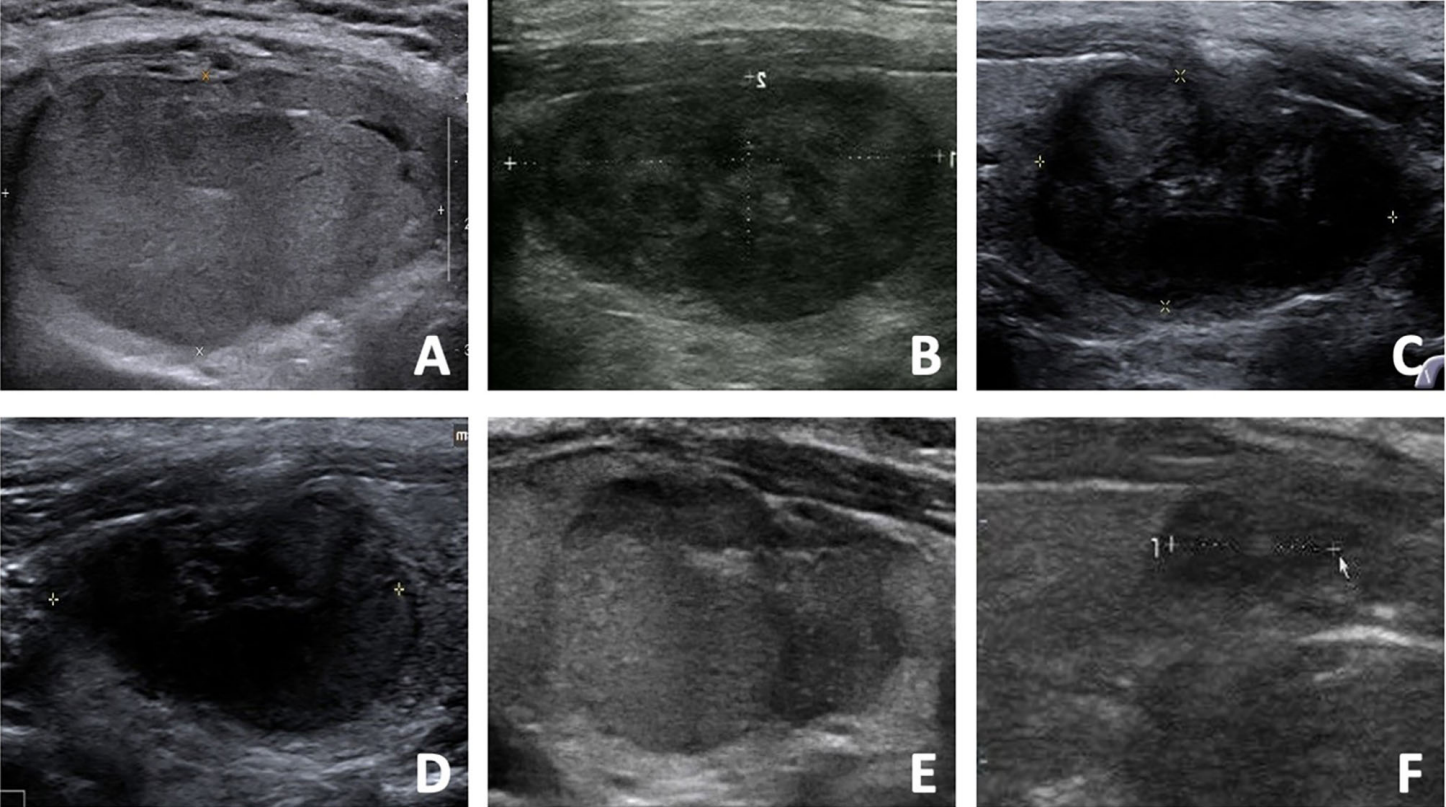
The US images of a 48- year-old male with a benign thyroid nodule in the regrowth group. **(A)** The initial volume of nodule was 22.27 ml before RFA. The nodule was located in the dangerous location, and the vascularity was grade 4. The risk of regrowth by the nomogram was 67%. **(B)** At 1 month after RFA, the volume was 5.28 ml and VRR was 76.29%. **(C)** At 3 months after RFA, the volume was 3.48 ml and VRR was 84.37%. **(D)** At 6 months after RFA, the volume was 2.89 ml and VRR was 87.02%. **(E)** At 12 months after RFA, the US showed nodule regrowth and the volume was 4.34 ml and VRR was 80.51%. Additional RFA was performed. **(F)** At 6 months after additional RFA, the volume was 1.65 ml and VRR was 92.59%.

**Figure 3:**
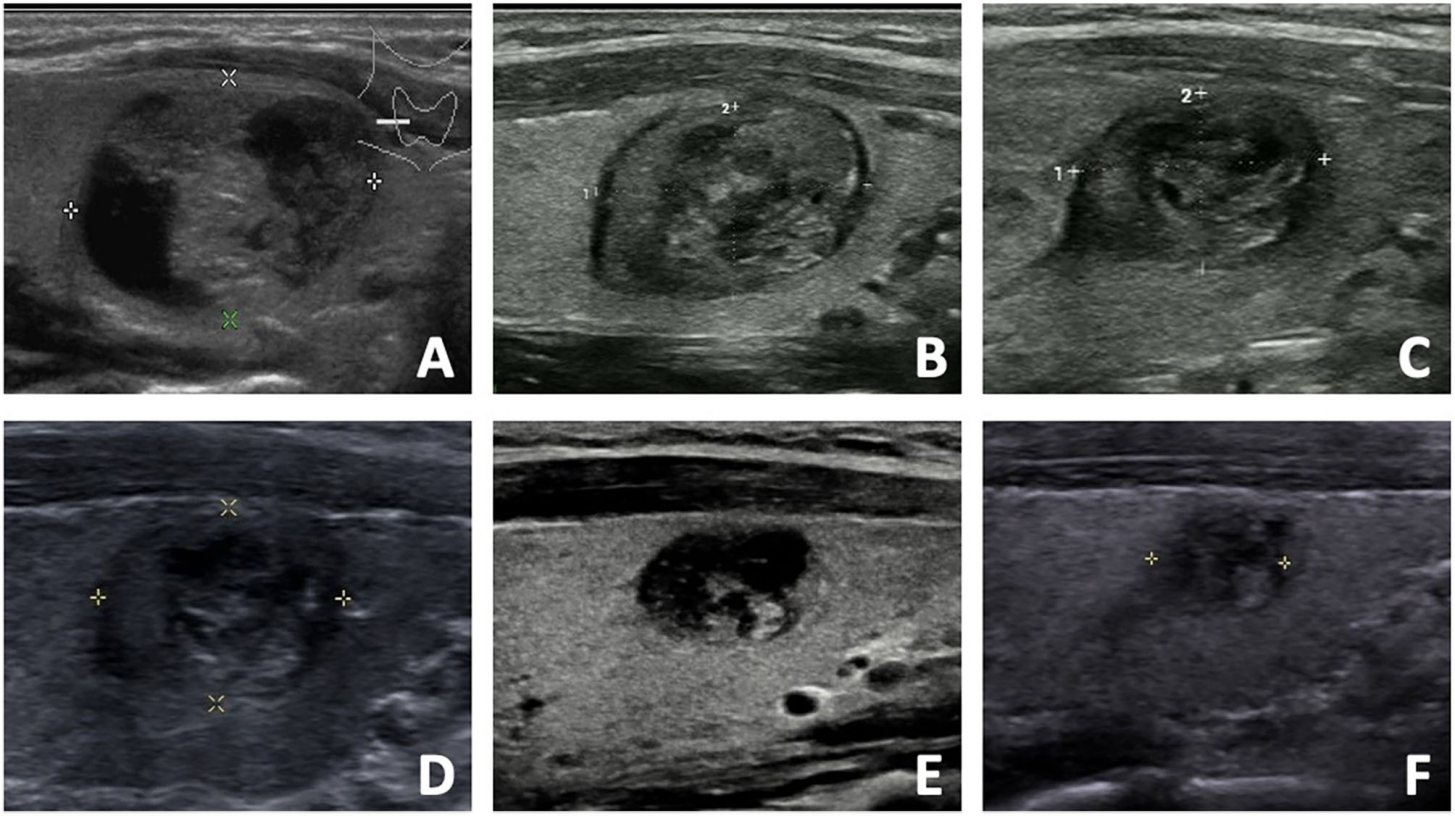
The US images of a 33- year-old female with a benign thyroid nodule in the non-regrowth group. **(A)** The initial volume of nodule was 5.32 ml before RFA. The nodule was located in the normal location, and the vascularity was grade 1. The risk of regrowth by the nomogram was 2.1%. **(B)** At 1 month after RFA, the volume was 1.58 ml and VRR was 70.30%. **(C)** At 3 months after RFA, the volume was 0.88 ml and VRR was 83.46%. **(D)** At 6 months after RFA, the volume was 0.76 ml and VRR was 85.71%. **(E)** At 12 months after RFA, the volume was 0.32 ml and VRR was 93.98%. **(F)** At 18 months after RFA, the volume was 0.18 ml and VRR was 96.62%.

Regrowth was observed in 30 out 220 nodules (13.64%), which all occurred in the untreated peripheral area. The mean timing of regrowth was at 22.40 ± 12.10 months after RFA. All the regrowth nodules underwent additional RFA. The changes of volume and VRR in the two groups are present in **Table 2**. In the first 12 months, VRR in the two groups were nonsignificant (all *P* >0.05). However, at 24 months after RFA, VRR in the non-regrowth group were significantly larger than that in the regrowth group (90.39 ± 11.23% vs 78.65 ± 12.24%, *P* = 0.002) (**Figure 1**). A total of 7 nodules had volume reduction less than 50% at 12 months. Five nodules were in the regrowth group and 2 in the non-regrowth group (16.67% vs 1.05%, *P* <0.001). Representative cases in the two groups are shown in **Figures 2**, **3**.

**Table 2:** Changes of volume and VRR at each follow-up period after RFA.

Time (months)	Volume (ml)				VRR (%)			
	Total	Non-regrowth group	Regrowth group	*P*-value	Total	Non-regrowth group	Regrowth group	*P*-value
1	4.89 ± 6.19	4.60 ± 6.22	6.86 ± 5.75	0.007	48.62 ± 19.63	48.26 ± 18.79	51.12 ± 25.03	0.350
3	2.72 ± 3.79	2.44 ± 3.54	4.54 ± 4.82	0.023	69.89 ± 16.61	69.59 ± 16.50	71.83 ± 17.60	0.394
6	2.60 ± 4.13	2.21 ± 3.44	4.65 ± 6.37	0.015	79.42 ± 13.44	79.22 ± 13.58	80.46 ± 12.95	0.595
12	2.06 ± 4.07	1.76 ± 3.36	4.42 ± 7.39	0.031	84.38 ± 14.07	84.92 ± 13.04	80.08 ± 20.47	0.489
24	1.90 ± 3.81	1.48 ± 3.13	4.60 ± 6.31	0.005	88.82 ± 11.98	90.39 ± 11.23	78.65 ± 12.24	0.002

Data are presented as mean ± SD.

The comparison of the two groups is shown in **Table 3**. The initial volume (19.33 ± 19.79 ml vs 8.88 ± 11.54 ml, *P* <0.001) and vascularity (3.0 ± 0.8 vs 2.4 ± 0.9, *P* = 0.002) in the regrowth group were significantly larger than those in the non-regrowth group. In the regrowth group, 33.67% (11/30) nodules were located close to the critical structure, while in the non-regrowth group 17.37% (33/190) of the nodules were close to the critical structure (*P* = 0.017). The energy and EPV in the regrowth group and non-regrowth group were nonsignificant (3,369.66 ± 2,281.05 J vs 2,527.84 ± 2,141.21 J, *P* = 0.070; 440.49 ± 684.83 J/ml vs 679.72 ± 673.78 J/ml, *P* = 0.085).

**Table 3:** The comparison between the non-regrowth and regrowth groups.

Characteristics	Non-regrowth group	Regrowth group	OR (95%CI)	*P*-value
Pre-treated variables				
Age (years)	46.84 ± 11.98	42.55 ± 11.35	0.970 (0.938–1.003)	0.074
Female	163 (85.8)	24 (80.0)	0.663 (0.248–1.771)	0.412
Solid	137 (72.11)	18 (60.00)	0.580 (0.262–1.287)	0.180
Initial volume (ml)	8.88 ± 11.54	19.33 ± 19.79	1.043 (1.019–1.068)	<0.001
Location close to critical structure	33 (17.37)	11 (33.67)	2.754 (1.199–6.330)	0.017
Hashimoto’s thyroiditis	34 (17.89)	3 (10)	0.510 (0.146–1.778)	0.290
Vascularity	2.4 ± 0.9	3.0 ± 0.8	2.161 (1.312–3.561)	0.002
Ablate-related variables				
Power (W)	6.88 ± 3.25	6.50 ± 2.01	0.957 (0.831–1.101)	0.535
Energy (J)	2,527.84 ± 2,141.21	3,369.66 ± 2,281.05	1.148 (0.989–1.333)	0.070
EPV (J/ml)	679.72 ± 673.78	440.49 ± 684.83	0.999 (0.998–1.000)	0.085

Data are presented as mean ± SD or number of tumors (percentages).

EPV, Energy per volume.

**Figure 4:**
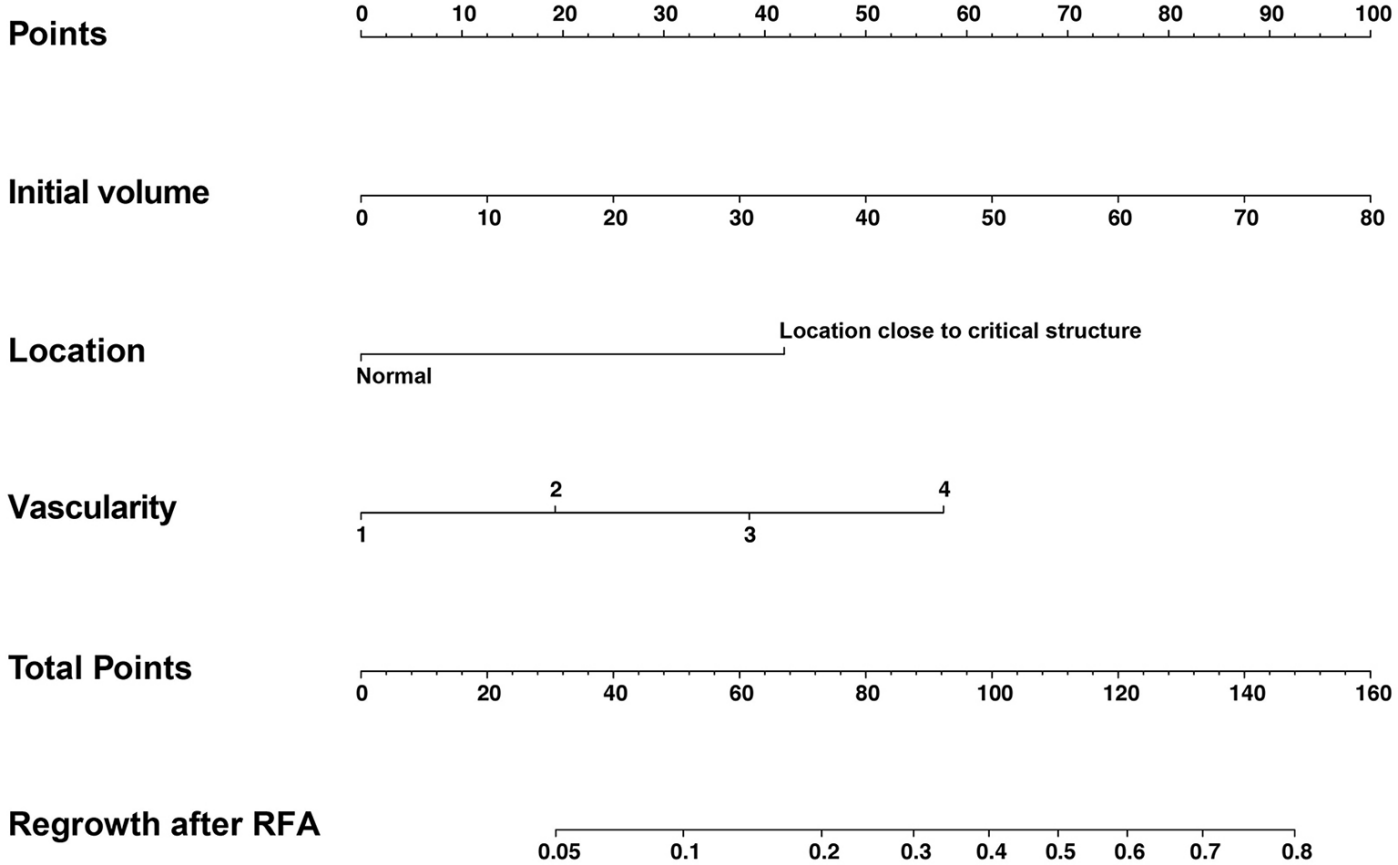
The nomogram for predicting regrowth after RFA for benign thyroid nodules. A nomogram based on initial volume, vascularity and location was constructed.

**Figure 5:**
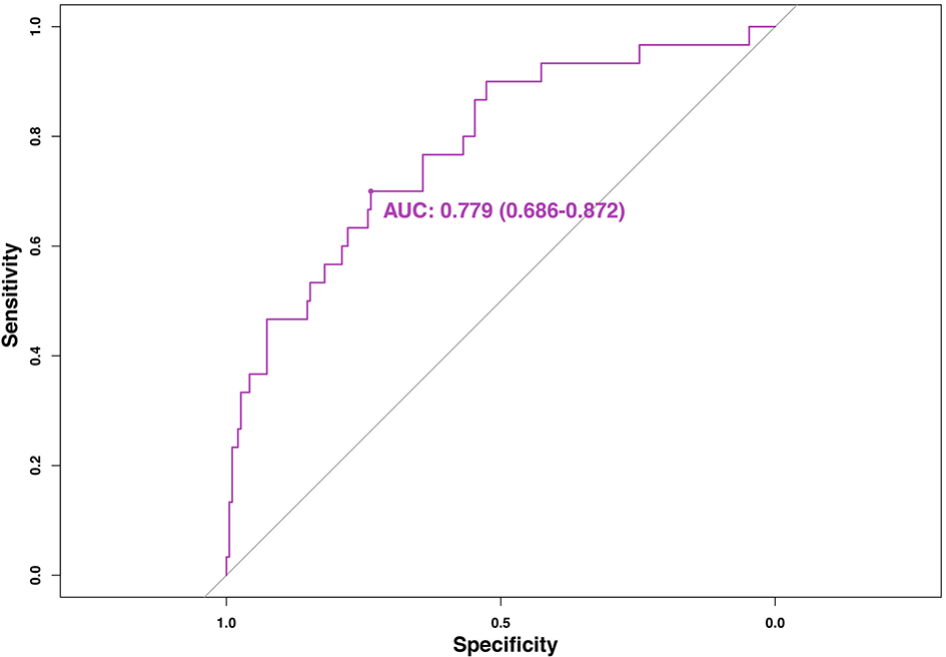
The ROC of the nomogram for predicting regrowth in patients with benign thyroid nodules after RFA. AUC for the nomogram to predict regrowth was 0.779 (95%CI 0.686–0.872).

**Figure 6:**
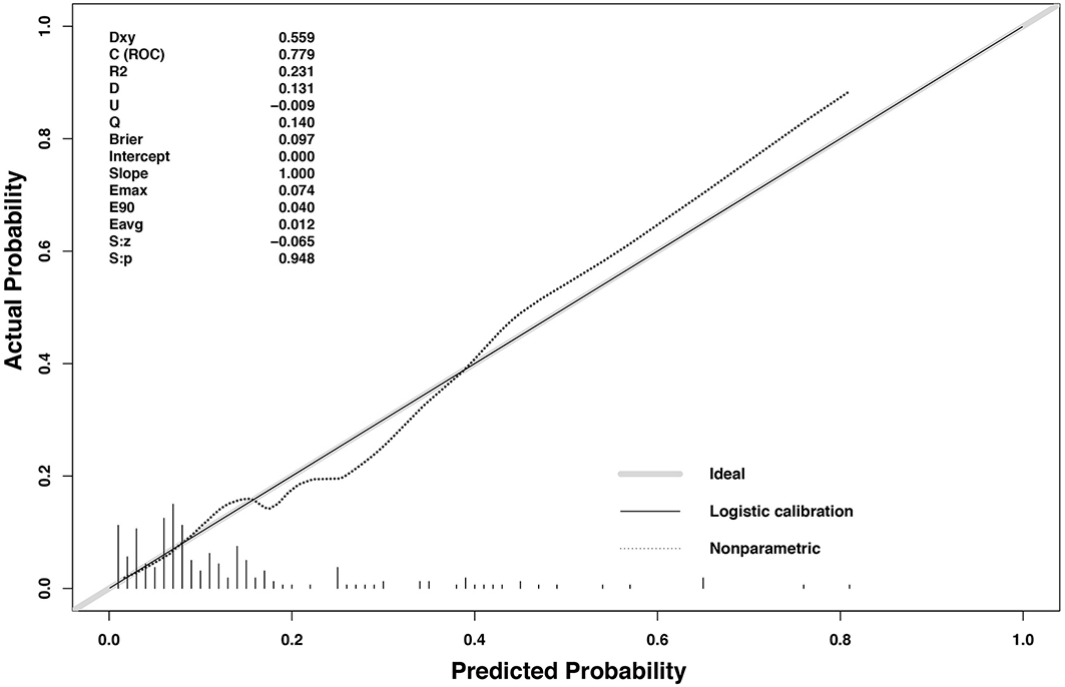
The calibration curves for predicting regrowth in patients with benign thyroid nodules after RFA. The x-axis represents the nomogram predicted probability and the y-axis represents the actual probability. The calibration curves were close to the 45° line. The diagonal dashed line indicates ideal prediction by a perfect model, and the solid line represents the predictive power of the nomogram. The closer the solid line is to the dotted line, the better is the predictive power of the model.

Multivariate logistic regression revealed initial volume (OR = 1.047, 95%CI 1.020–1.075), vascularity (OR = 2.037, 95%CI 1.218–3.404) and location close to critical structure (OR = 4.713, 95%CI 1.817–12.223) were independent factors associated with regrowth (**Table 4**). A nomogram based on these three independent factors to predict nodule regrowth after RFA was constructed (**Figure 4**). Each factor was allocated a predicting score, and the sum of three scores was located on the total points axis, suggesting the prediction of regrowth probabilities. Higher total points were associated with a higher regrowth probability during the follow-up. The discriminative ability of the model for regrowth was assessed using the C-index, which was 0.779 (95%CI 0.686–0.872) (**Figure 5**). The accuracy of the model and potential model overfit were assessed by bootstrap validation with 1,000 re-samplings. The calibration curves graphically showed good agreement between the actual and nomogram-predicted regrowth (**Figure 6**).

**Table 4:** Multivariate logistic regression analysis of regrowth.

Characteristics	OR	95%CI	*P*-value
Initial volume (ml)	1.047	1.020–1.075	<0.001
Location close to critical structure	4.713	1.817–12.223	0.001
Vascularity	2.037	1.218–3.404	0.007

No major complications occurred during or after RFA. Eighteen patients (9.00%) had local pain or discomfort and resolved spontaneously within 1 week.

## Discussion

No prediction model for regrowth after thermal ablation for benign thyroid nodules has been reported to date. This study developed and validated a nomogram to predict regrowth after RFA for benign thyroid nodules. The C-index of this nomogram was 0.779 with a good calibration, which suggested that the nomogram could successfully stratified patients before treatment according to their risk of regrowth and yielded excellent performance. This model would be helpful to guide decision-making and provide individualized ablation management.

As alternatives to surgery for benign thyroid nodules by guidelines (4–6), the ablation efficacy has attracted much attention. The volume reduction after ablation was significant with a low rate of complications (17, 18, 21). However, the effective treatment of ablation should be sustainable for longer follow-up period. Some studies showed a tendency for the nodules to regrow during the follow-up and the reported incidence of regrowth after RFA, MWA and LA was 5.6–24.1% (19–21), 14.55% (22) and 37.8–38% (21, 23), respectively. Several predictors were reported to be associated with regrowth, but the optimal prediction of regrowth was still unclear. Sim et al. (20) found that the total volume of nodule after ablation could be divided into Va and Vv. The results found that Vv increase which was defined as a more than 50% increase compared to the previously reported smallest Vv, occurred earlier than regrowth and might be used as an early sign (20). However, Vv increase was observed at 27.5 ± 18.5 months after RFA, making its predictive value limited. Negro et al. (23) reported that a 12-month VRR <50% increased the risk of regrowth after LA with an OR of 11.7 (95%CI 4.2–32.2). However, compared with LA, the efficacy of RFA or MWA seemed to be much better with a higher volume reduction (17, 37–40) and a lower regrowth rate (19–23). The predictive value of 12-month VRR of these two ablation techniques still needed further investigation. Furthermore, whether EPV could affect ablation efficacy or regrowth remained controversial. A higher EPV was found to be associated with the efficacy of ablation (21, 41, 42). Wang et al. (22) found that EPV in the non-regrowth group was significantly larger than that in the regrowth group after MWA. However, contradictory results were also observed. Negro et al. (28) found that EPV was significantly larger in the nodules with 12-month VRR <50% than that in nodules with 12-month VRR ≥50%, indicating the former were not treated with an insufficient amount of energy. In this study, although EPV in the non-regrowth group were larger than that in the regrowth group, no significant differences were found. The reason of different results might be related to the diverse thermal ablation techniques, nodule structure and volume, which could lead to different production and distribution of thermal energy (21), making the predict value of EPV still uncertain.

CLT, also known as Hashimoto thyroiditis, is an autoimmune disease that destroys thyroid cells by cell and antibody-mediated immune process. The pathology of the disease involves the formation of antithyroid antibodies that attack the thyroid tissue, causing widespread lymphocyte infiltration, fibrosis, and parenchymal atrophy of the thyroid tissue. This study found that CLT was not an independent factor related to regrowth by multivariate analysis. It was consistent with previous study (43), which reported that the recovery after RFA in patients with PTMC + CLT was similar to that in patients with only PTMC. It suggested that although the thyroid parenchyma in the case of CLT was already infiltrated by diffuse chronic inflammatory cells, it did not influence the clinical outcomes of RFA for benign thyroid nodules.

In this study, a nomogram incorporated three easily identifiable variables was developed to predict nodule regrowth after RFA. The performance was well supported by the C-index of 0.779 and the calibration curves demonstrating the optimal agreements between prediction and actual observation, which guaranteed the repeatability and reliability of the established nomogram. To the best of our knowledge, it was the first nomogram to predict nodule regrowth after ablation, which could be helpful to improve the patient-physician communication, decision-making and individualized treatment management. Though this easy-to-use scoring system, physicians could perform an individualized prediction before ablation to identify patients at different risk of regrowth. Patients with a high score had an increased probability of nodule regrowth and were potential candidates for additional treatment. Therefore, these patients needed to be provided appropriate ablation strategy and intensive follow-up management or recommended surgery if they do not want multiple sessions.

So far, no consensus about the specific indication or optimal timing of additional ablation for benign thyroid nodules was achieved (43). In most studies, additional ablation was usually determined based on clinical evaluation findings, namely, incompletely symptom resolution, or unsatisfactory volume reduction, or regrowth (4). Sim et al. (20) suggested that additional ablation might be performed after the detection of Vv increase because it was important to identify regrowth. A recent study compared the efficacy of additional RFA after different indications. Additional RFA performed after Vv increased revealed superior efficacy, including a significant greater volume reduction improvement of cosmetic and symptom scores (44). It was because that the residual volume was markedly larger when Vv increase was observed. Additional ablation could be applied to a larger residual zone and achieve substantial incremental volume reduction. Therefore, to maximize the efficacy of ablation, additional RFA could be considered after the detection of Vv increase.

This study found that initial volume, location close to critical structure and vascularity were independent factors related to regrowth. Initial volume has been recognized as an important factor for regrowth (19, 20, 43). It might be difficult to ablate all the nodule margin of a large nodule by a single session, leading to incomplete treatment and subsequent regrowth. Location was another factor associated with regrowth (22). The neck was relatively narrow, where contained many critical structures. As a result, complications might be inevitable when a nodule located close to the critical structures. For hyper-vascular nodules, the possibility of regrowth also existed (22). The vasculature in the thyroid nodules could cause heat-sink effect, which decreased the ablation efficacy and induced regrowth (45). To obtain complete ablation, several techniques have been recommended, namely, the moving shot technique, the hydrodissection technique, and vascular ablation techniques (4). The moving shot technique has been suggested as a suitable method for the peripheral ablation. This technique could treat the nodule completely and safely by dividing it into multiple small ablation units (45). Moreover, the hydrodissection technique was effective to separate the nodule from the adjacent critical structures (45, 46). By injecting the fluid into the naturally existed gap between the cervical anatomical structures, a protective thermal barrier was formed to minimize the complications and improve the efficacy. Additionally, vascular ablation techniques have been introduced as an advanced technique for hyper-vascular nodules (45). The main feeding artery was ablated first followed by the draining vein along the nodule margin, which could not only damage the blood vessels and decrease the heat-sink effect, but also prevent incomplete ablation of the peripheral area.

This study had some limitations. First, because of the retrospective nature, sample bias was existed. Moreover, some potentially variables were not available in the data set and thus could not be included in this nomogram, such as the experience of US physician, different ablation techniques and inflammatory response caused by ablation. A large prospective study is needed to further confirm the reliability of the nomogram. Second, although this nomogram was internally validated using bootstrap validation, the clinical use must be externally validated and evaluated. Third, the follow-up was relatively short. There were two peaks of nodule regrowth (20). First peak of regrowth ranged from 1 year to 2 or 3 years, and the second one appeared later than 5 years (20). The predictive value of this nomogram on the second peak of regrowth is still needed further investigation.

## Conclusion

This study constructed a nomogram to predict regrowth in patients with benign thyroid nodules after RFA with good discrimination and calibration capabilities. The use of this prognostic nomogram may guide physician in stratifying patients and provide precise guidance for individualized treatment protocols.

## Data Availability Statement

The original contributions presented in the study are included in the article/supplementary files, further inquiries can be directed to the corresponding authors.

## Ethics Statement

The studies involving human participants were reviewed and approved by the Institutional Review Board of Chinese PLA General Hospital. The patients/participants provided their written informed consent to participate in this study.

## Author Contributions

YL interpreted the patient data and drafted the manuscript. LYK performed RFA procedure, conceived of the study and coordination. MBZ, XYL, YYL collected and analyzed the patient data. All authors contributed to the article and approved the submitted version.

## Funding

This study is supported by the Popularization of Military Medical and Health Achievements (No. 21WKS09).

## Conflict of Interest

The authors declare that the research was conducted in the absence of any commercial or financial relationships that could be construed as a potential conflict of interest.

## Publisher’s Note

All claims expressed in this article are solely those of the authors and do not necessarily represent those of their affiliated organizations, or those of the publisher, the editors and the reviewers. Any product that may be evaluated in this article, or claim that may be made by its manufacturer, is not guaranteed or endorsed by the publisher.

## References

[1] Durante C Grani G Lamartina L Filetti S SJ M Cooper DS. The Diagnosis and Management of Thyroid Nodules: A Review. JAMA (2018) 319(9):914–24. doi: 10.1001/jama.2018.0898 29509871

[2] Gharib H Papini E Paschke R Duick DS Valcavi R Hegedus L. American Association of Clinical Endocrinologists, Associazione Medici Endocrinologi, and European Thyroid Association Medical Guidelines for Clinical Practice for the Diagnosis and Management of Thyroid Nodules. J Endocrinol Invest (2010) 33(5 Suppl):1–50. doi: 10.4158/10024.GL 20543550

[3] Haugen BR Alexander EK Bible KC Doherty GM Mandel SJ Nikiforov YE. 2015 American Thyroid Association Management Guidelines for Adult Patients With Thyroid Nodules and Differentiated Thyroid Cancer: The American Thyroid Association Guidelines Task Force on Thyroid Nodules and Differentiated Thyroid Cancer. Thyroid (2015) 26(1):1–133. doi: 10.1089/thy.2015.0020 PMC4739132 26462967

[4] Kim JH Baek JH Lim HK Ahn HS Baek SM Choi YJ. 2017 Thyroid Radiofrequency Ablation Guideline: Korean Society of Thyroid Radiology. Korean J Radiol (2018) 19(4):632–55. doi: 10.3348/kjr.2018.19.4.632 PMC6005940 29962870

[5] Papini E Pacella CM Solbiati LA Achille G Barbaro D Bernardi S. Minimally-Invasive Treatments for Benign Thyroid Nodules: A Delphi-Based Consensus Statement From the Italian Minimally-Invasive Treatments of the Thyroid (MITT) Group. Int J Hyperthermia (2019) 36(1):376–82. doi: 10.1080/02656736.2019.1575482 30909759

[6] Dietrich CF Müller T Bojunga J Dong Y Mauri G Radzina M. Statement and Recommendations on Interventional Ultrasound as a Thyroid Diagnostic and Treatment Procedure. Ultrasound Med Biol (2018) 44(1):14–36. doi: 10.1016/j.ultrasmedbio.2017.08.1889 29126752

[7] Garberoglio R Aliberti C Appetecchia M Attard M Boccuzzi G Boraso F. Radiofrequency Ablation for Thyroid Nodules: Which Indications? The First Italian Opinion Statement. J Ultrasound (2015) 18(4):423–30. doi: 10.1007/s40477-015-0169-y PMC4630279 26550079

[8] Deandrea M Sung JY Limone P Mormile A Garino F Ragazzoni F. Efficacy and Safety of Radiofrequency Ablation Versus Observation for Nonfunctioning Benign Thyroid Nodules: A Randomized Controlled International Collaborative Trial. Thyroid (2015) 25(8):890–6. doi: 10.1089/thy.2015.0133 26061686

[9] Cesareo R Pasqualini V Simeoni C Sacchi M Saralli E Campagna G. Prospective Study of Effectiveness of Ultrasound-Guided Radiofrequency Ablation Versus Control Group in Patients Affected by Benign Thyroid Nodules. J Clin Endocrinol Metab (2015) 100(2):460–6. doi: 10.1210/jc.2014-2186 25387256

[10] Hamidi O Callstrom MR Lee RA Dean D Castro MR Morris JC. Outcomes of Radiofrequency Ablation Therapy for Large Benign Thyroid Nodules: A Mayo Clinic Case Series. Mayo Clin Proc (2018) 93(8):1018–25. doi: 10.1016/j.mayocp.2017.12.011 29572016

[11] Radzina M Cantisani V Rauda M Nielsen MB Ewertsen C D’Ambrosio F. Update on the Role of Ultrasound Guided Radiofrequency Ablation for Thyroid Nodule Treatment. Int J Surg (2017) 41:S82–93. doi: 10.1016/j.ijsu.2017.02.010 28506420

[12] Rabuffi P Spada A Bosco D Bruni A Vagnarelli S Ambrogi C. Treatment of Thyroid Nodules With Radiofrequency: A 1-Year Follow-Up Experience. J Ultrasound (2019) 22(2):193–9. doi: 10.1007/s40477-019-00375-4 PMC6531559 30945239

[13] Kotewall N Lang BHH. High-Intensity Focused Ultrasound Ablation as a Treatment for Benign Thyroid Diseases: The Present and Future. Ultrasonography (2019) 38(2):135–42. doi: 10.14366/usg.18040 PMC6443589 30690961

[14] Bernardi S Dobrinja C Carere A Giudici F Calabrò V Zanconati F. Patient Satisfaction After Thyroid RFA Versus Surgery for Benign Thyroid Nodules: A Telephone Survey. Int J Hyperthermia (2018) 35(1):150–8. doi: 10.1080/02656736.2018.1487590 30107758

[15] Lang BHH Woo YC Chiu KW. Two-Year Efficacy of Single-Session High-Intensity Focused Ultrasound (HIFU) Ablation of Benign Thyroid Nodules. Eur Radiol (2019) 29(1):93–101. doi: 10.1007/s00330-018-5579-8 29922925

[16] Lin WC Kan NN Chen HL Luo SD Tung YC Chen WC. Efficacy and Safety of Single-Session Radiofrequency Ablation for Benign Thyroid Nodules of Different Sizes: A Retrospective Study. Int J Hyperthermia (2020) 37(1):1082–9. doi: 10.1080/02656736.2020.1782485 32964743

[17] Trimboli P Castellana M Sconfienza LM Virili C Pescatori LC Cesareo R. Efficacy of Thermal Ablation in Benign Non-Functioning Solid Thyroid Nodule: A Systematic Review and Meta-Analysis. Endocrine (2019) 67(1):35–43. doi: 10.1007/s12020-019-02019-3 31327158

[18] Chung SR Suh CH Baek JH Park HS Choi YJ Lee JH. Safety of Radiofrequency Ablation of Benign Thyroid Nodules and Recurrent Thyroid Cancers: A Systematic Review and Meta-Analysis. Int J Hyperthermia (2017) 33(8):920–30. doi: 10.1080/02656736.2017.1337936 28565997

[19] Lim HK Lee JH Ha EJ Sung JY Kim JK Baek JH. Radiofrequency Ablation of Benign Non-Functioning Thyroid Nodules: 4-Year Follow-Up Results for 111 Patients. Eur Radiol (2013) 23(4):1044–9. doi: 10.1007/s00330-012-2671-3 23096937

[20] Sim JS Baek JH Lee J Cho W Jung SI. Radiofrequency Ablation of Benign Thyroid Nodules: Depicting Early Sign of Regrowth by Calculating Vital Volume. Int J Hyperthermia (2017) 33(8):905–10. doi: 10.1080/02656736.2017.1309083 28540795

[21] Bernardi S Giudici F Cesareo R Antonelli G Cavallaro M Deandrea M. Five-Year Results of Radiofrequency and Laser Ablation of Benign Thyroid Nodules: A Multicenter Study From the Italian Minimally Invasive Treatments of the Thyroid Group. Thyroid (2020) 30(12):1759–70. doi: 10.1089/thy.2020.0202 32578498

[22] Wang B Han ZY Yu J Cheng Z Liu F Yu XL. Factors Related to Recurrence of the Benign Non-Functioning Thyroid Nodules After Percutaneous Microwave Ablation. Int J Hyperthermia (2017) 33(4):459–64. doi: 10.1080/02656736.2016.1274058 28081645

[23] Negro R Greco G Deandrea M Rucco M Trimboli P. Twelve-Month Volume Reduction Ratio Predicts Regrowth and Time to Regrowth in Thyroid Nodules Submitted to Laser Ablation: A 5-Year Follow-Up Retrospective Study. Korean J Radiol (2020) 21(6):764–72. doi: 10.3348/kjr.2019.0798 PMC7231608 32410415

[24] Mauri G Pacella CM Papini E Solbiati L Goldberg SN Ahmed M. Image-Guided Thyroid Ablation: Proposal for Standardization of Terminology and Reporting Criteria. Thyroid (2019) 29(5):611–8. doi: 10.1089/thy.2018.0604 30803397

[25] Dossing H Bennedbaek FN Hegedus L. Long-Term Outcome Following Interstitial Laser Photocoagulation of Benign Cold Thyroid Nodules. Eur J Endocrinol (2011) 165(1):123–8. doi: 10.1530/eje-11-0220 21551168

[26] Valcavi R Riganti F Bertani A Formisano D Pacella CM. Percutaneous Laser Ablation of Cold Benign Thyroid Nodules: A 3-Year Follow-Up Study in 122 Patients. Thyroid (2010) 20(11):1253–61. doi: 10.1089/thy.2010.0189 20929405

[27] Yan L Luo Y Xie F Zhang M Xiao J. Residual Vital Ratio: Predicting Regrowth After Radiofrequency Ablation for Benign Thyroid Nodules. Int J Hyperthermia (2020) 37(1):1139–48. doi: 10.1080/02656736.2020.1825835 32996790

[28] Negro R Rucco M Creanza A Mormile A Limone PP Garberoglio R. Machine Learning Prediction of Radiofrequency Thermal Ablation Efficacy: A New Option to Optimize Thyroid Nodule Selection. Eur Thyroid J (2020) 9(4):205–12. doi: 10.1159/000504882 PMC7445654 32903883

[29] Iasonos A Schrag D Raj GV Panageas KS. How to Build and Interpret a Nomogram for Cancer Prognosis. J Clin Oncol (2008) 26(8):1364–70. doi: 10.1200/JCO.2007.12.9791 18323559

[30] Hu H Han H Han XK Wang WP Ding H. Nomogram for Individualised Prediction of Liver Failure Risk After Hepatectomy in Patients With Resectable Hepatocellular Carcinoma: The Evidence From Ultrasound Data. Eur Radiol (2018) 28(2):877–85. doi: 10.1007/s00330-017-4900-2 28779402

[31] Wang Y Li J Xia Y Gong R Wang K Yan Z. Prognostic Nomogram for Intrahepatic Cholangiocarcinoma After Partial Hepatectomy. J Clin Oncol (2013) 31(9):1188–95. doi: 10.1200/jco.2012.41.5984 23358969

[32] Levy DA Li H Sterba KR Hughes-Halbert C Warren GW Nussenbaum B. Development and Validation of Nomograms for Predicting Delayed Postoperative Radiotherapy Initiation in Head and Neck Squamous Cell Carcinoma. JAMA Otolaryngol Head Neck Surg (2020) 146(5):455–64. doi: 10.1001/jamaoto.2020.0222 PMC7118672 32239201

[33] An C Li X Yu X Cheng Z Han Z Liu F. Nomogram Based on Albumin-Bilirubin Grade to Predict Outcome of the Patients With Hepatitis C Virus-Related Hepatocellular Carcinoma After Microwave Ablation. Cancer Biol Med (2019) 16(4):797–810. doi: 10.20892/j.issn.2095-3941.2018.0486 31908896 PMC6936230

[34] Ni JY Fang ZT Sun HL An C Huang ZM Zhang TQ. A Nomogram to Predict Survival of Patients With Intermediate-Stage Hepatocellular Carcinoma After Transarterial Chemoembolization Combined With Microwave Ablation. Eur Radiol (2020) 30(4):2377–90. doi: 10.1007/s00330-019-06438-8 31900694

[35] Huang Z Gu Y Zhang T Wu S Wang X An C. Nomograms to Predict Survival Outcomes After Microwave Ablation in Elderly Patients (>65 Years Old) With Early-Stage Hepatocellular Carcinoma. Int J Hyperthermia (2020) 37(1):808–18. doi: 10.1080/02656736.2020.1785556 32619374

[36] Kao WY Su CW Chiou YY Chiu NC Liu CA Fang KC. Hepatocellular Carcinoma: Nomograms Based on the Albumin-Bilirubin Grade to Assess the Outcomes of Radiofrequency Ablation. Radiology (2017) 285(2):670–80. doi: 10.1148/radiol.2017162382 28562211

[37] Shi YF Zhou P Zhao YF Liu WG Tian SM Liang YP. Microwave Ablation Compared With Laser Ablation for Treating Benign Thyroid Nodules in a Propensity-Score Matching Study. Front Endocrinol (Lausanne) (2019) 10:org/10.3389/fendo.2019.00874. doi: 10.3389/fendo.2019.00874 PMC6923173 31920983

[38] Ha EJ Baek JH Kim KW Pyo J Lee JH Baek SH. Comparative Efficacy of Radiofrequency and Laser Ablation for the Treatment of Benign Thyroid Nodules: Systematic Review Including Traditional Pooling and Bayesian Network Meta-Analysis. J Clin Endocrinol Metab (2015) 100(5):1903–11. doi: 10.1210/jc.2014-4077 25695887

[39] Cesareo R Manfrini S Pasqualini V Ambrogi C Sanson G Gallo A. Laser Ablation Versus Radiofrequency Ablation for Thyroid Nodules: 12-Month Results of a Randomized Trial (LARA II Study). J Clin Endocrinol Metab (2021) 106(6):1692–701. doi: 10.1210/clinem/dgab102 33608728

[40] Cesareo R Pacella CM Pasqualini V Campagna G Iozzino M Gallo A. Laser Ablation Versus Radiofrequency Ablation for Benign Non-Functioning Thyroid Nodules: Six-Month Results of a Randomized, Parallel, Open-Label, Trial (LARA Trial). Thyroid (2020) 30(6):847–56. doi: 10.1089/thy.2019.0660 32056501

[41] Deandrea M Trimboli P Mormile A Cont AT Milan L Buffet C. Determining an Energy Threshold for Optimal Volume Reduction of Benign Thyroid Nodules Treated by Radiofrequency Ablation. Eur Radiol (2021) 31(7):5189–97. doi: 10.1007/s00330-020-07532-y 33409792

[42] Trimboli P Deandrea M. Treating Thyroid Nodules by Radiofrequency: Is the Delivered Energy Correlated With the Volume Reduction Rate? A Pilot Study. Endocrine (2020) 69(3):682–7. doi: 10.1007/s12020-020-02275-8 32319012

[43] Zhang Y Zhang MB Luo YK Li J Zhang Y Tang J. Effect of Chronic Lymphocytic Thyroiditis on the Efficacy and Safety of Ultrasound-Guided Radiofrequency Ablation for Papillary Thyroid Microcarcinoma. Cancer Med (2019) 8(12):5450–8. doi: 10.1002/cam4.2406 PMC6746112 31359613

[44] Sim JS Baek JH. Long-Term Outcomes Following Thermal Ablation of Benign Thyroid Nodules as an Alternative to Surgery: The Importance of Controlling Regrowth. Endocrinol Metab (Seoul) (2019) 34(2):117–23. doi: 10.3803/EnM.2019.34.2.117 PMC6599899 31257739

[45] Yan L Luo Y Zhang M Xiao J. Vital Volume Increase Versus Clinical Evaluation as the Indication of Additional Radiofrequency Ablation for Benign Thyroid Nodule: A Single Center Retrospective Study. Int J Hyperthermia (2020) 37(1):777–85. doi: 10.1080/02656736.2020.1778197 32619366

[46] Park HS Baek JH Park AW Chung SR Choi YJ Lee JH. Thyroid Radiofrequency Ablation: Updates on Innovative Devices and Techniques. Korean J Radiol (2017) 18(4):615–23. . doi: 10.3348/kjr.2017.18.4.615 PMC5447637 28670156

